# Automatic Identification of Highly Conserved Family Regions and Relationships in Genome Wide Datasets Including Remote Protein Sequences

**DOI:** 10.1371/journal.pone.0075458

**Published:** 2013-09-12

**Authors:** Tunca Doğan, Bilge Karaçalı

**Affiliations:** 1 Biotechnology and Bioengineering Graduate Program, Izmir Institute of Technology, Izmir, Turkey; 2 Institute of Health Sciences, Dokuz Eylul University, Izmir, Turkey; 3 Electrical and Electronics Engineering Department, Izmir Institute of Technology, Izmir, Turkey; University of South Florida College of Medicine, United States of America

## Abstract

Identifying shared sequence segments along amino acid sequences generally requires a collection of closely related proteins, most often curated manually from the sequence datasets to suit the purpose at hand. Currently developed statistical methods are strained, however, when the collection contains remote sequences with poor alignment to the rest, or sequences containing multiple domains. In this paper, we propose a completely unsupervised and automated method to identify the shared sequence segments observed in a diverse collection of protein sequences including those present in a smaller fraction of the sequences in the collection, using a combination of sequence alignment, residue conservation scoring and graph-theoretical approaches. Since shared sequence fragments often imply conserved functional or structural attributes, the method produces a table of associations between the sequences and the identified conserved regions that can reveal previously unknown protein families as well as new members to existing ones. We evaluated the biological relevance of the method by clustering the proteins in gold standard datasets and assessing the clustering performance in comparison with previous methods from the literature. We have then applied the proposed method to a genome wide dataset of 17793 human proteins and generated a global association map to each of the 4753 identified conserved regions. Investigations on the major conserved regions revealed that they corresponded strongly to annotated structural domains. This suggests that the method can be useful in predicting novel domains on protein sequences.

## Introduction

Large amounts of data regarding the molecular attributes of living organisms are being accumulated in databases since the availability of molecular scanning tools over the last decades. A considerable amount of this data consists of molecular sequences.

Biomolecular sequences carry the information that make up the whole structural properties and metabolism of an organism. As each one has a specific task in the life of the organism, they are highly diverse. Yet, some of them still share sequential features. These features emerge as statistically significant similarities between different regions of the sequences, often indicating mutuality in the history of these sequences and/or commonality in metabolic functions. This information is an important instrument for the discovery of the molecular mechanisms that govern the organism’s system. These shared regions are grouped under two widely used terms in the literature; motifs and domains.

A motif is a short sequence fragment usually composed of just a few nucleotides or amino acids that possess biological significance, while a domain is a part of a protein sequence that can function, evolve and fold independent from the rest of the protein [1], [2]. Protein domains are up to 600 amino acids in length and usually highly specific. Each domain has a specific molecular function and a protein’s functional attributes are directly related to the domains it contains. Domains are collected in vast databases under different names, subject to varying rules and regulations, such as Protein families (Pfam) [3], InterPro [4], NCBI Conserved Domain Database [5], SCOP: Structural Classification of Proteins [6], CATH Protein Structure Classification [7] and Simple Modular Architecture Research Tool (SMART) [8].

Motifs and domains are believed to be highly conserved during the evolutionary process since changes in their primary structure can cause the loss of vital molecular functions. In order to extract these highly conserved/shared sequential features, biomolecular sequences are compared and contrasted to each other using efficient sequence analysis methods that also invoke concepts from graph theory. In these methods, biomolecular sequences are treated as vertices of a graph, and a connection between two vertices represents the presence of a significant statistical sequential similarity between the corresponding sequences.

GeneRAGE [9] and TRIBE-MCL [10] are two of the earliest methods to employ this concept in similarity-based methods. TRIBE-MCL incorporates Markov Clustering for rapid and accurate clustering of proteins especially addressing multi-domain sequences. Apeltsin *et al.*, add an edge weight distribution along with an automated threshold selection to initial similarity network, and increase the clustering performance of fast MCL to match novel highly efficient clustering algorithms on a gold standard dataset [11]. Spectral Clustering [12], another Markov Clustering algorithm with a global approach also offered with a user-interface SCPS [13], is an efficient and widely used algorithm for sequence clustering.

The Connected Component Analysis is another widely used graph theory application [14], employed both as a stand-alone method and as an intermediary step in other sequence clustering methods. Briefly, in an undirected graph *G*, two vertices *a* and *b* are connected if there is a path from *a* to *b*. A connected component is defined as a connected sub-graph of *G* whose vertices are pairwise connected to each other (directly or indirectly) [14]. Sequences in a connected component are then presumed to share a significant sequence similarity, and thus, belong to the same cluster of sequences. On the other hand, when the Connected Component Analysis is used on multi-domain proteins, unrelated sequences can group within the same cluster due to the domain-chaining effect [15].

The Cluster-C [15] method efficiently avoids the chaining effect by incorporating maximal clique extraction on the connectivity map following a pairwise similarity search. However, the incorporation of maximal clique finding into clustering suffers from practical problems especially on large datasets, such as clique redundancy. In theory, sequences in each maximal clique should contain at least one unique conserved feature. In practice, though, the maximal cliques are redundant, with a shared region represented in more than one maximal clique. This arises from the accidental removal of pairwise connections due to remote homology or just poor alignment between some of the homolog sequence pairs.

Three important bottle-necks stand out in general in motif discovery approaches. The first one is the treatment of multi-domain proteins: Most of these methods are optimized to process single domain sequences and the assignment of multi-domain proteins into clusters is often problematic. The second issue is the standardization of the input parameters: The behaviors of these algorithms are controlled by several parameters to be provided by the user at the input stage. However, in the absence of a known standard to deduce the optimal parameters given the input sequences, selecting the correct parameters becomes nearly impossible. As a result, the accuracy of the results becomes questionable. Finally, in most of the methods, no further processing can be performed for the remote input sequences left out as singleton points after the initial similarity search.

In this paper, we propose a new method named CRIS (acronym for Conserved Region Identification and Search) for the automatic allocation of diverse sequences into biologically relevant groups by exposing highly conserved regions and associating them with the input sequences using statistical grouping and graph theory concepts. The method is completely unsupervised requiring no information except the sequences at the input level. This is done first by grouping the sequences in connected components characterized by significant sequence similarities based on pairwise alignment e-values, and then, splitting the sequences in each connected component into maximal cliques consisting of sequences containing a shared feature. Next, the shared/conserved regions on multiple sequence alignments of the member sequences of each maximal clique were identified using a residue conservation scoring algorithm, and conserved region profiles were generated and subsequently queried on the input sequences. Finally, the associations between the input sequences and the identified highly conserved regions were presented as a table that can be used to infer structural, functional and/or evolutionary relationships between the sequences.

We have tested the proposed method’s biological relevance by carrying out clustering on gold standard sequence datasets from the SCOP Database [6] that were used previously in the literature [12], [13] and comparing clustering performances to the widely used clustering methods.

We have also applied the proposed method on a genome wide dataset of 17793 human protein sequences to obtain a global familial relation map of human proteins. The dataset contained both similar and considerably distant proteins. We have evaluated the performance of the proposed method in the identification of the documented domains on the input sequences by comparing the identified conserved/shared regions and their associations with the input sequences to the reference manually curated domain associations obtained from Protein Family (Pfam) database [3] and NCBI Conserved Domain Database (CDD) [16]. The results revealed that the discovered conserved regions highly correspond to documented domains on the input proteins.

The details of the proposed conserved region discovery and association method are presented in the next section. The results of the comparative performance evaluation experiments as well as the application of the method to 17793 human proteins is provided in Results, followed by the discussion of the results along with the significance of the method in Discussion.

## Materials and Methods

**Figure 1:**
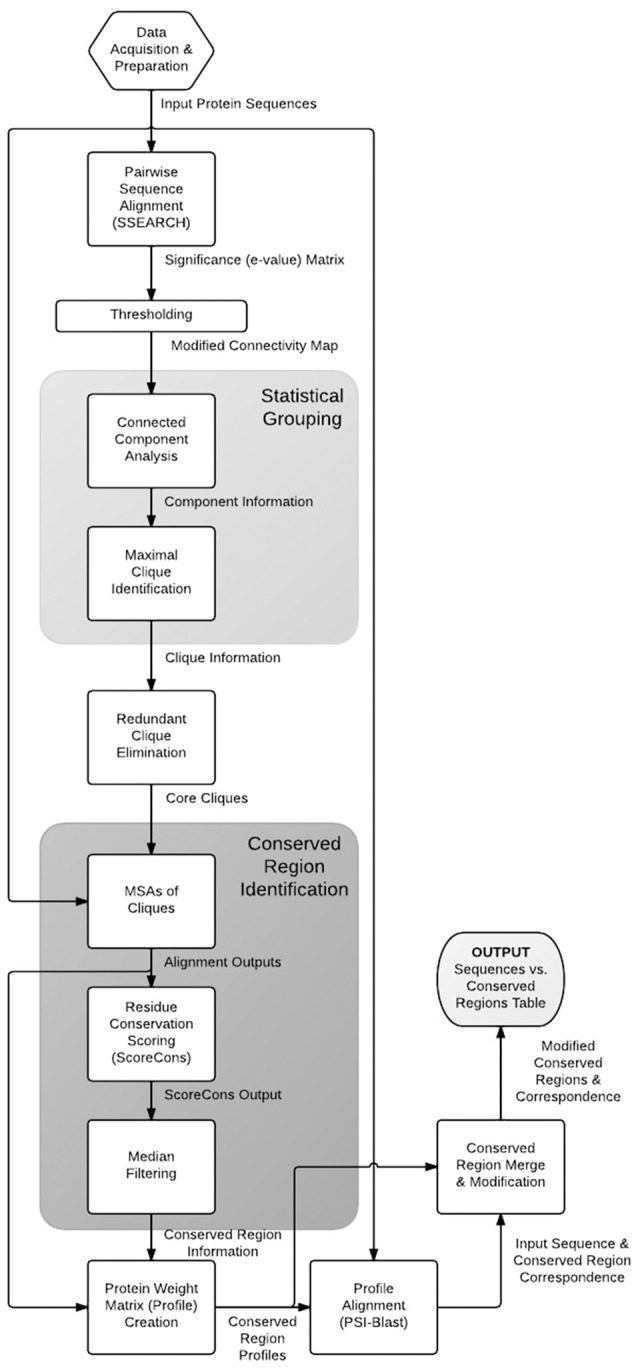
Flow diagram of the proposed method. Flow diagram of the proposed method.

The flow diagram of the proposed method is given in Figure 1. We describe each step in detail below.

### Pairwise Sequence Alignment

A stand-alone version of SSEARCH algorithm from FASTA v36.3.5 software package [17] was used for the Smith-Waterman pairwise all-against-all sequence alignment [18] with the default options. This choice represented the preference for alignment accuracy at the expense of computation time, though the BLAST algorithm [19] could also be used albeit with a preference for the reduced computational expense. Running time for the all-against-all pairwise alignment step was O(*n* ^2^) where *n* represents the number of input sequences.

After that, a pairwise connectivity map was formed using the pairwise alignment e-values threshold with 0.01. The pairwise similarities with e-values higher than the threshold are discarded. The threshold here is selected to be fairly loose in order to only remove very weak similarities/connections. The purpose behind the removal of the weak connections was the elimination of the deduced similarity between the sequences with poor pairwise alignment. This specific threshold value was selected after many trial tests on reference datasets (data not shown), though it is possible to set a different threshold value to suit a particular sequence dataset.

### Statistical Grouping

Routines provided by the MATLAB® Bioinformatics Toolbox (The MathWorks Inc., 2010) were used for the Connected Component Analysis using the binary connectivity map generated at the previous step as the input. The input sequences were grouped into components possessing a direct or an indirect connection between every sequence pair. The running time for the connected component analysis step was O(*N*+*E*) where *N* represents the number of nodes and *E* represents the number of edges. Due to the domain chaining effect, some of the diverse sequences are expected to be clustered together at the end of the Connected Component Analysis especially in the case of analyzing the multi-domain proteins.

**Figure 2:**
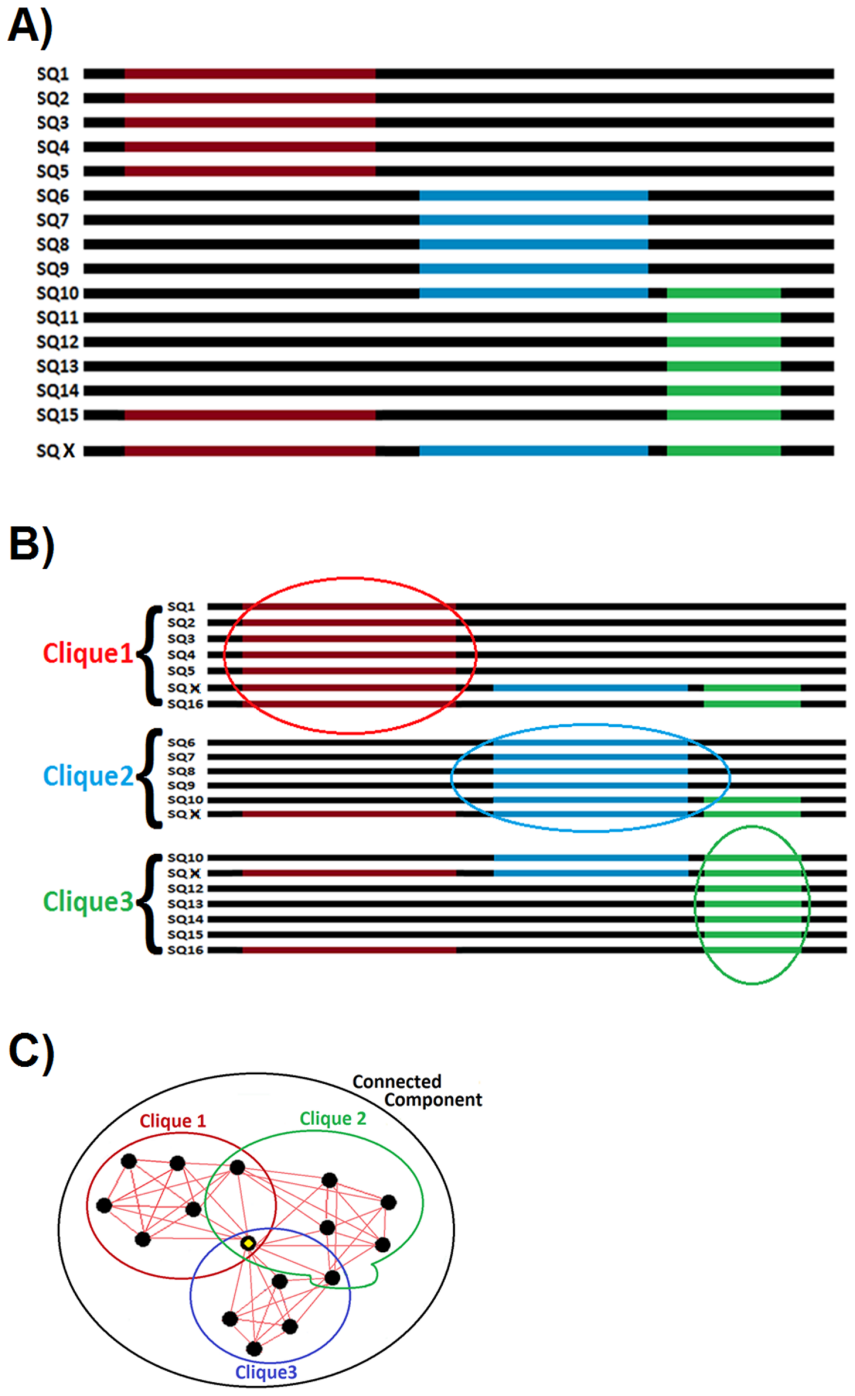
Statistical grouping. Representation of the statistical grouping procedure.

Next, the maximum clique finding operation was applied separately on the members of each connected component using the Bron–Kerbosch algorithm [20]. A maximal clique, or a fully connected sub-component, is a subset of an undirected graph where each vertex is directly connected to every other vertex. This means that all proteins in a maximal clique share a unique sequential feature (a conserved sequence segment). Note that unlike connected components, a vertex (sequence) may appear in more than one maximal clique. This allows capturing a second, third or so on features located on a sequence by looking at its involvement in different maximal cliques. The part A of Figure 2 shows 15 protein sequences with the different family regions highlighted in different colors and the sequence of a multi-domain/family protein (SQ X) with 3 different consecutively located family regions at the last row. The part B of the figure shows the clusters of sequences after the statistical grouping process. The part C of the figure shows the two-dimensional undirected graph representation of the results. The black dots represent the different sequences and the red lines represent the edges corresponding to statistically significant similarities. Finally, the large black circle indicates the connected component. Here, all sequences belong to a single component since there is either a direct or an in-direct connection between them. Colored circles represent the three maximal cliques. The vertex marked by yellow corresponds to the sequence shared between all cliques, the last sequence in part A (SQ X).

The worst-case running time for the maximal clique identification step was O(3*^n^* ^/3^) where *n* is the number of sequences in each connected component. The NP-complete nature of the maximal clique finding operation presented computational challenges for the connected components possessing a large number of sequences. To address this issue, the connected components with more than 100 sequences were randomly divided into groups of 100 sequences and the maximal clique identification procedure was applied on these groups separately. Supposing there is a connected component with 850 sequences, it will be split into 9 groups (8 groups of 100 sequences and 1 group of 50 sequences). In order to detect the families with more than 100 member sequences, a later step was employed in the procedure involving the merging of the redundant conserved regions.

Note that the non-homologous sequences that were potentially brought together at the previous step are separated from each other by the maximal clique identification process since a full inter-connection between the sequences is required to be clustered within the same clique.

As a result of the maximal clique identification procedure, several redundant cliques were produced that differed from each other by a few sequences, revolving around an underlying clique missing a few connections in the connectivity map. In order to detect and eliminate the redundant cliques, Hamming distances [21] between maximal clique pairs were computed. The Hamming distance is a measure of difference between two strings of equal length, counting the number of substitutions to change the first string into the second [21]. We defined the fractional Hamming distance between a pair of cliques as the regular Hamming distance divided by the total number of sequences in both cliques. This normalization eliminated the effects of the possible discrepancy between the clique sizes on the distance measure. Calculation of the fractional Hamming distance is given in Equation 1.
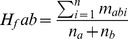
(1)


In the expression above, *H_fab_* is the fractional Hamming distance between cliques *a* and *b*, *m_abi_* is a binary variable that represent the match or the mismatch at the *i* ^th^ position between cliques *a* and *b,* equalling 0 if there is a match and 1 if there is a mismatch, *n* is the total number of proteins in the test, *n_1_* and *n_2_* are the number of proteins in the corresponding cliques.

**Figure 3:**
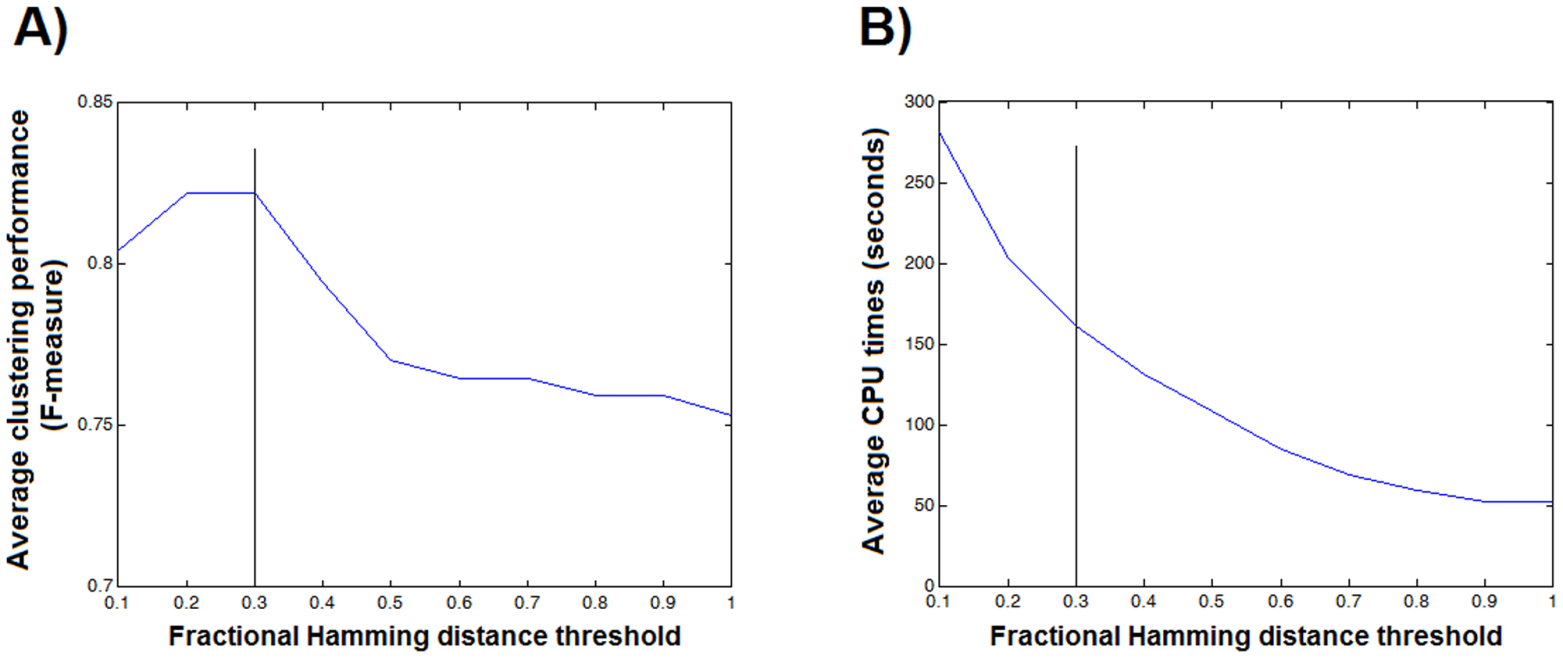
Optimization of the fractional Hamming distance threshold. Plots for the optimization of the fractional Hamming distance threshold: (A) the average clustering performances (F-measure) vs. fractional Hamming distances, (B) average CPU times (in seconds) vs. fractional Hamming distances.

The cliques were then clustered using a pre-defined fractional Hamming distance threshold of 0.3 and the redundant cliques were eliminated by selecting the clique with the highest number of sequences to represent each group. Figure 3 shows the selection of the fractional Hamming distance threshold regarding the performance of the method in the clustering of reference SCOP datasets. Part A of the figure shows the average clustering performances assessed by the F-measure for varying fractional Hamming distance thresholds. In part B, average CPU times (in seconds) to run the process on a 2.3 GHz single core and 50 GB of RAM is plotted against varying fractional Hamming distance thresholds. The vertical black lines on both plots indicate the selected threshold. At 0.3, the maximum clustering performance was obtained within a reasonable CPU time. The running time for the redundant clique elimination step was O(*n* ^2^) where *n* represents the number of maximal cliques.

### Conserved Region Identification & Search Process

First, member proteins of each individual maximal clique were subjected to global multiple sequence alignment using the ClustalO package [22] with the default parameters. The use of the procedure here is quite standard that any multiple sequence alignment algorithm (for general purpose) would do the necessary job. Clustal family tools are among the most widely known alignment algorithms for general use. With such a well-known general-purpose alignment technique, we desired to maintain general applicability of the method, whose performance could otherwise have been attributed to a less well-known and more specialized alignment tool had we opted for one. Among the Clustal family methods, ClustalO was used as it provides the parallelization of the process on multiple cores, reducing the CPU times significantly. The running time for the conserved region identification (alignment step) was O(*n*) where *n* represents the number of maximal cliques.

Following the alignment step, the ScoreCons algorithm [23], a residue conservation scoring method was employed with the default parameters to review the multiple alignment of each maximal clique. Many different residue conservation scoring methods exist in the literature. These methods are designed to scan the multiple alignments column-wise and reveal the conservation degree of each position in terms of the stereochemical diversity, diversity of symbols based on theoretical entropy, and/or amino acid frequency [23]. In this work, the valdar01 scoring method [23] was used, where a substitution matrix was employed to evaluate the stereochemical diversity. Consequently, each position was scored between 0 that indicated no conservation and 1, indicating full conservation. This procedure allows the identification of highly conserved regions (highlighted with colors in Figure 2, part B) on the multiple sequence alignment outputs of the members of each maximal clique. The running time for the conserved region identification (residue conservation scoring step) was O(n*P) where *n* is the number of maximal cliques and *P* is the number of positions in each alignment.

**Figure 4:**
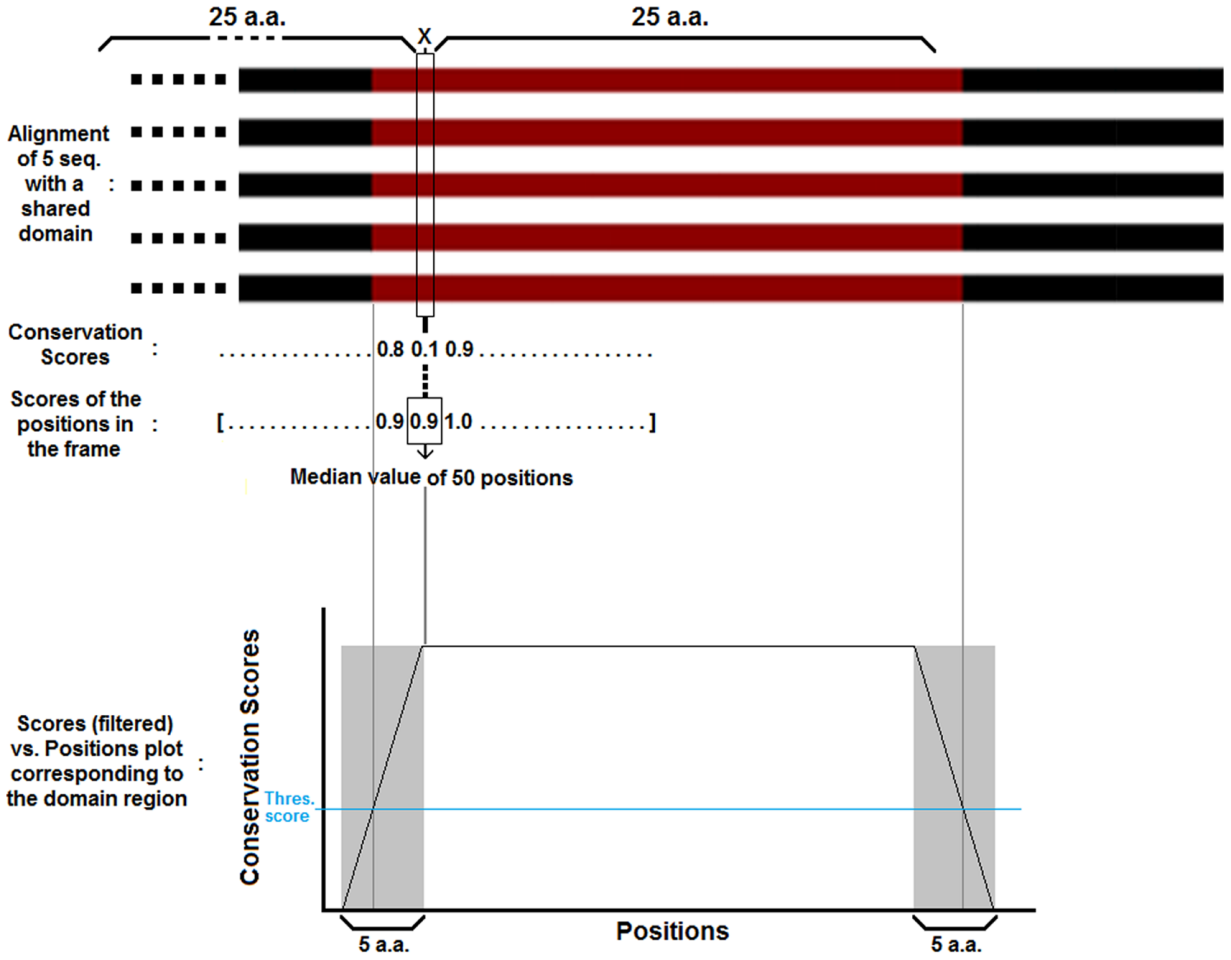
Median filtering. Representation of the Median filtering application to the residue conservation scores.

Since the residue conservation scoring algorithm acts on each position independently, the output was inevitably noisy. In order to clearly identify the conserved regions, we have used the one-dimensional median filtering method [24] with a neighborhood or frame size of 50 to filter out this noise. This method was shown to preserve the edges in the original signal better than most of the linear de-noising/smoothing methods [24], and yield a more accurate detection around the boundaries. The frame size of the median filter was set to match the minimum size of the conserved regions: This was aimed at detecting the conserved regions that are longer than 20 amino acids, since nearly all of the structural domains registered on the databases currently fall in this range. Figure 4 shows a segment of an alignment of 5 sequences sharing a family region during the application of median filtering for smoothing the residue conservation scores. The row below the alignment shows the raw conservation scores. The position marked by an “X” is being processed in the case where the raw conservation score is only 0.1 although the position is inside the family region. With a frame size of 50, the raw conservation scores of 25 positions to the right and 25 to the left are taken together with the score of the position X and these 51 values are sorted in an increasing order. The median value (the 26^th^ value in the ordered list) is assigned to the position X. In the example, this value is 0.9. The plot of the median filtered curve is shown at the bottom side of the figure. The horizontal blue line represents the threshold score for the positions to be accepted as part of a conserved region.

Note that since the conservation around the boundaries of family regions is often less prominent, there is a probability of error of a few residues (about ±2.5) for misplacing the boundaries. Since the frame size is 50 in the median filtering operation, highly conserved regions shorter than 25 residues will be filtered out completely. The shortest possible detected region size can thus be 20 residues; when the original family region is 25 residues long and the boundary positions are rounded off by the filter possibly reducing the region by another 5 residues. The running time for the conserved region identification (score filtering step) was O(n*P) where *n* is the number of maximal cliques and *P* is the number of positions in each alignment.

Another key component here is the selection of the threshold conservation score to determine the conserved positions. In principle, the threshold should strike a balance to identify only the true conserved residues without missing any. In order to determine such a threshold, we have downloaded reference manually curated multiple sequence alignments of different eukaryotic proteins that were used for building NCBI-curated domain profiles [16]. The locations of the domains on these alignments were known in advance. Following the application of the residue conservation scoring algorithm on the reference alignments and the subsequent median filtering, the threshold score providing the most accurate identification of the domains was determined via statistical analysis.

**Figure 5:**
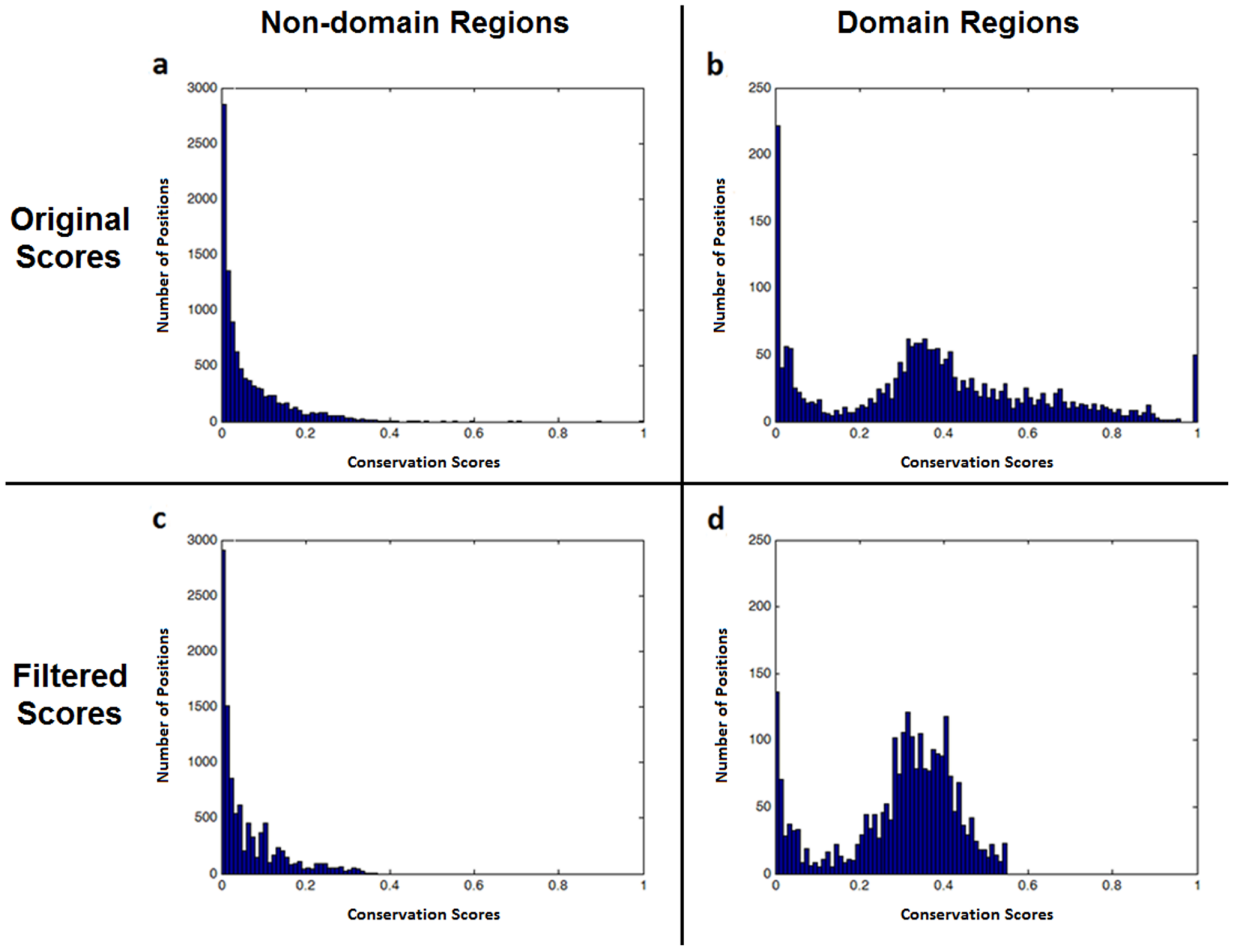
Residue conservation scoring histograms. Residue conservation scoring histograms of curated multiple sequence alignments of different eukaryotic proteins. The original scores: (a) the residues outside the NCBI-curated domains (non-domain regions), (b) the residues inside the NCBI-curated domains (domain regions). Filtered scores: (c) non-domain regions (d) domain regions.

**Figure 6:**
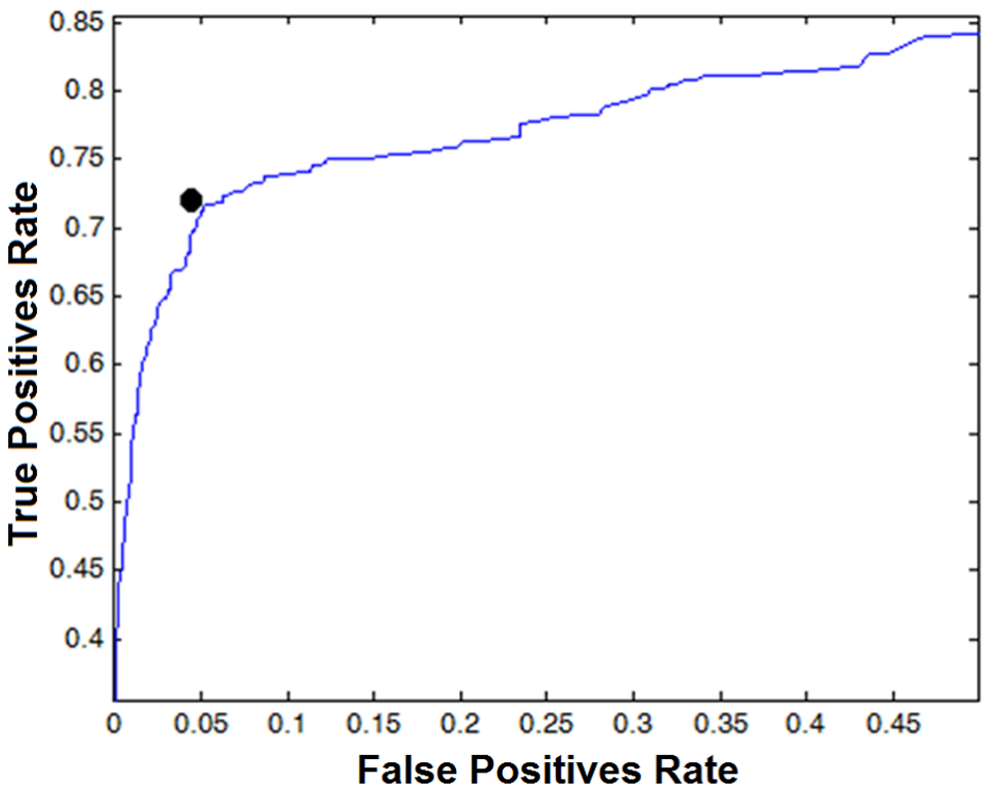
The ROC curve. The ROC curve for the binary classification of residues of reference multiple sequence alignments as domain or non-domain regions for the determination of threshold score in residue conservation scoring process. The black dot indicates the TPR and FPR values at the selected threshold.

Histograms of the residue conservation scores of domain and non-domain regions on original scores and on smoothed/filtered scores are shown in Figure 5. Clearly, higher scores accumulated conspicuously in the domain regions. The filtering operation reduced the number of the low scoring positions within domain regions by nearly 30%. To determine the optimal threshold conservation score, a receiver operating characteristic (ROC) curve [25] was drawn using reference labels of all positions, calculating the true positives rate (sensitivity) and the false positives rate (fall-out) for varying threshold selections (Figure 6). The optimum point at the knee of the ROC curve was determined to be equal to 0.2. The positions that have a conservation score over 0.2 were thus taken to be conserved and a conserved/shared region was subsequently formed by an uninterrupted series of conserved positions no shorter than 20 amino acids.

**Figure 7:**
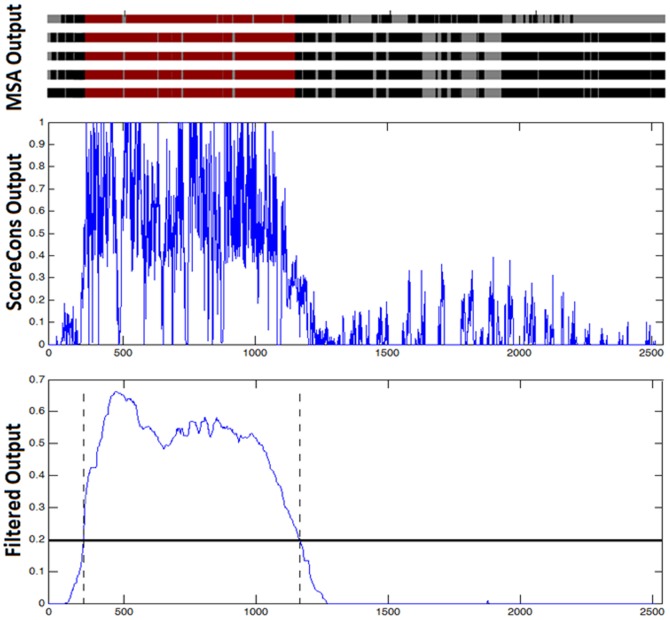
The complete conserved region identification process. Top: Multiple sequence alignment output of members of a sample clique, middle: residue conservation scoring process (ScoreCons) output, bottom: smoothed output with Median filtering, horizontal black line: threshold score to assume conservation, vertical dashed line: the borders of the recovered conserved region.

Figure 7 shows the complete conserved region identification process. On the top, an actual multiple sequence alignment output of the members of a sample clique from the human protein dataset is shown, where each row represents a different sequence. Red regions represent a shared domain/family region on these proteins, black regions are the remaining filled positions and the gray ones are the gaps. The plot in the middle shows the residue conservation scoring output on this alignment, with the elevated conservation scores corresponding to the domain region. The output of the median filtering operation is shown on the bottom plot. The positions with scores higher than the optimal conservation threshold at 0.2 formed the conserved region. Note also that a nearly perfect correspondence is obtained between the identified conserved region and the reference domain/family region.

Profiles consisting of the frequency of amino acids as well as the gaps were created for all conserved/shared regions based on the conserved region identification results. These profiles were then aligned to all sequences in the dataset using a local version of Position Specific Iterative Blast (PSI-blast) algorithm [19] with the default parameters. PSI-blast takes a query sequence, searches through a database, forms a profile (a PSSM) with the query and the significant hits, and searches the database once again, this time querying the profile to include more remote hits. This procedure then repeats iteratively until convergence. As a result, remote homologs are retrieved that might be missed with a normal blast search [19]. The queries in our case were the previously generated conserved region profiles, calling for the execution of the PSI-blast using the “querying an intermediate PSSM” option. In order to include only highly significant hits, we have used a threshold of 10^−5^ over the e-values and carried out the algorithm only for one iteration. The running time for the conserved region search step was O(n*N) where *n* is the number of input sequences and *N* is the number of conserved regions. For the example in Figure 2 part B, with the completion of the conserved region search process, all of the conserved regions can accurately be identified on SQ X.

### Conserved Region Merge and Modification

This operation was applied on the collection of conserved regions identified above, to remove the potential duplicate conserved regions coming from the redundant maximal cliques that may still be remaining. To this end, non-gapped consensus sequences of conserved region profiles were generated and aligned to each other in an all-against-all manner using a Smith-Waterman pairwise local alignment procedure [18] provided by the SSEARCH algorithm from FASTA v36.3.5 software package [17] with the default options.

**Figure 8:**
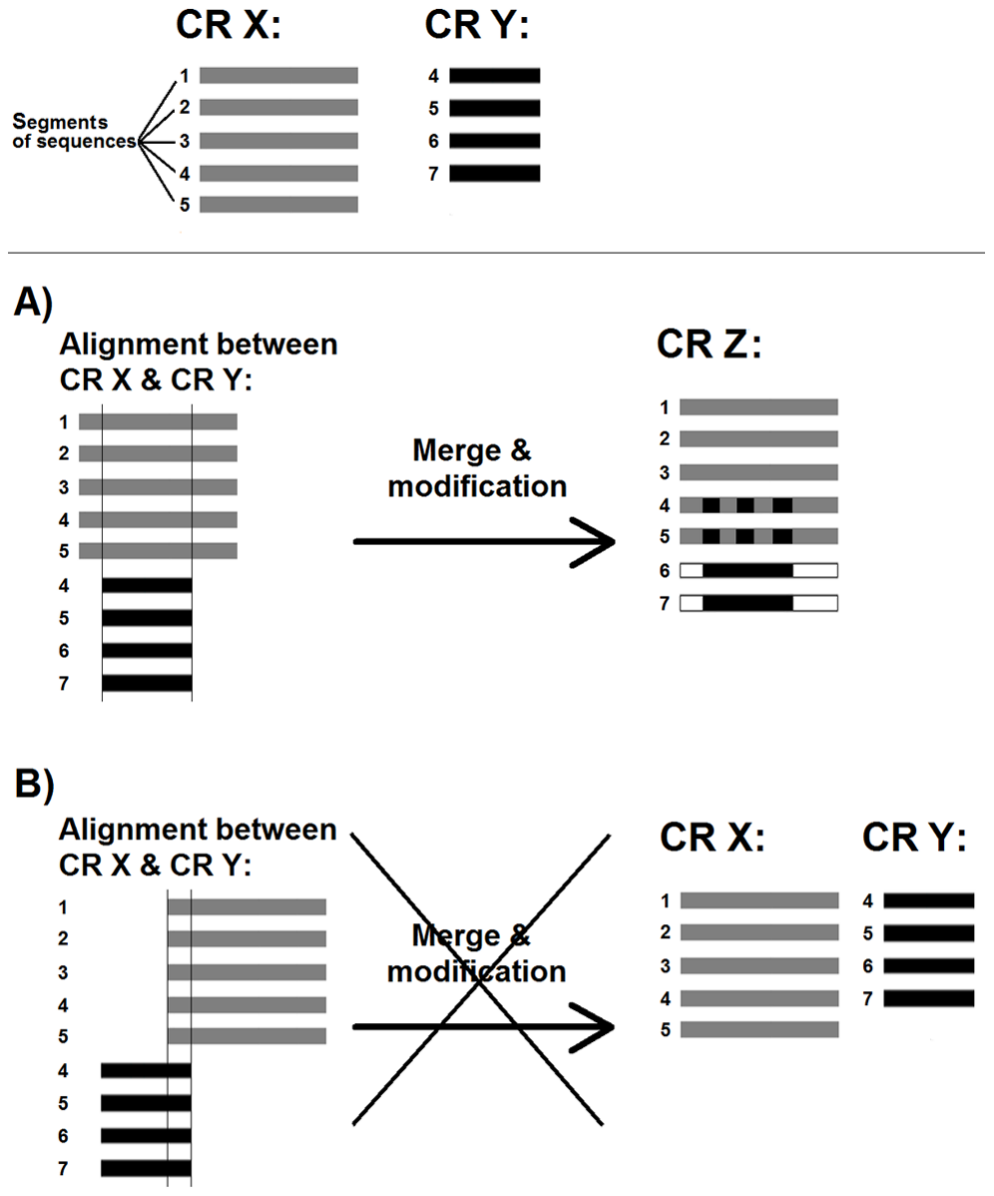
Conserved region merge and modification. Representation of the conserved region merge and modification procedure.

Specifically, among the significantly aligned (e-value < 0.01) conserved region pairs, if one was contained in the other or the overlap between them was greater than 90% they were assumed to represent the same family and merged into a new conserved region. The profile of the new conserved region was generated by combining the amino acid counts in the aligned profiles. The protein sequences associated with the new conserved region were determined by the union of those associated with the two original conserved regions. In cases where the above conditions were not satisfied, the conserved regions were not merged. Figure 8 shows two significantly aligned conserved regions X and Y where their associated sequences are 1, 2, 3, 4, 5 and 4, 5, 6, 7 respectively. Part A of the figure shows the case where the shorter one is contained within the longer one as identified by the pairwise alignment. These two conserved regions were merged into a new conserved region (Z) with the combined sequence associations of 1, 2, 3, 4, 5, 6 and 7. Part B of the figure shows another instance where the two conserved regions have a slight overlap, in which case the merging operation is not carried out.

The members of the same families separated from each other accidently at the random grouping to reduce the computational expense (before the maximal clique finding procedure) and therefore clustered in different cliques were also accurately identified and brought together at this step. This way, the method can reliably detect the domain families with more than 100 members. As a result, the remaining conserved regions contained unique attributes distinct from each other. The running time for the conserved region merge and modification step was O(*n* ^2^) where *n* is the number of conserved regions.

Finally, a binary table was generated that represented the identified conserved regions along its columns and the input sequences along its rows, with ones and zeros indicating the presence or absence of the conserved regions in each sequence. This table and the profiles of the identified conserved regions constituted the main output of the proposed method.

### Performance evaluation of the method in the prediction of domains/family regions

**Figure 9:**
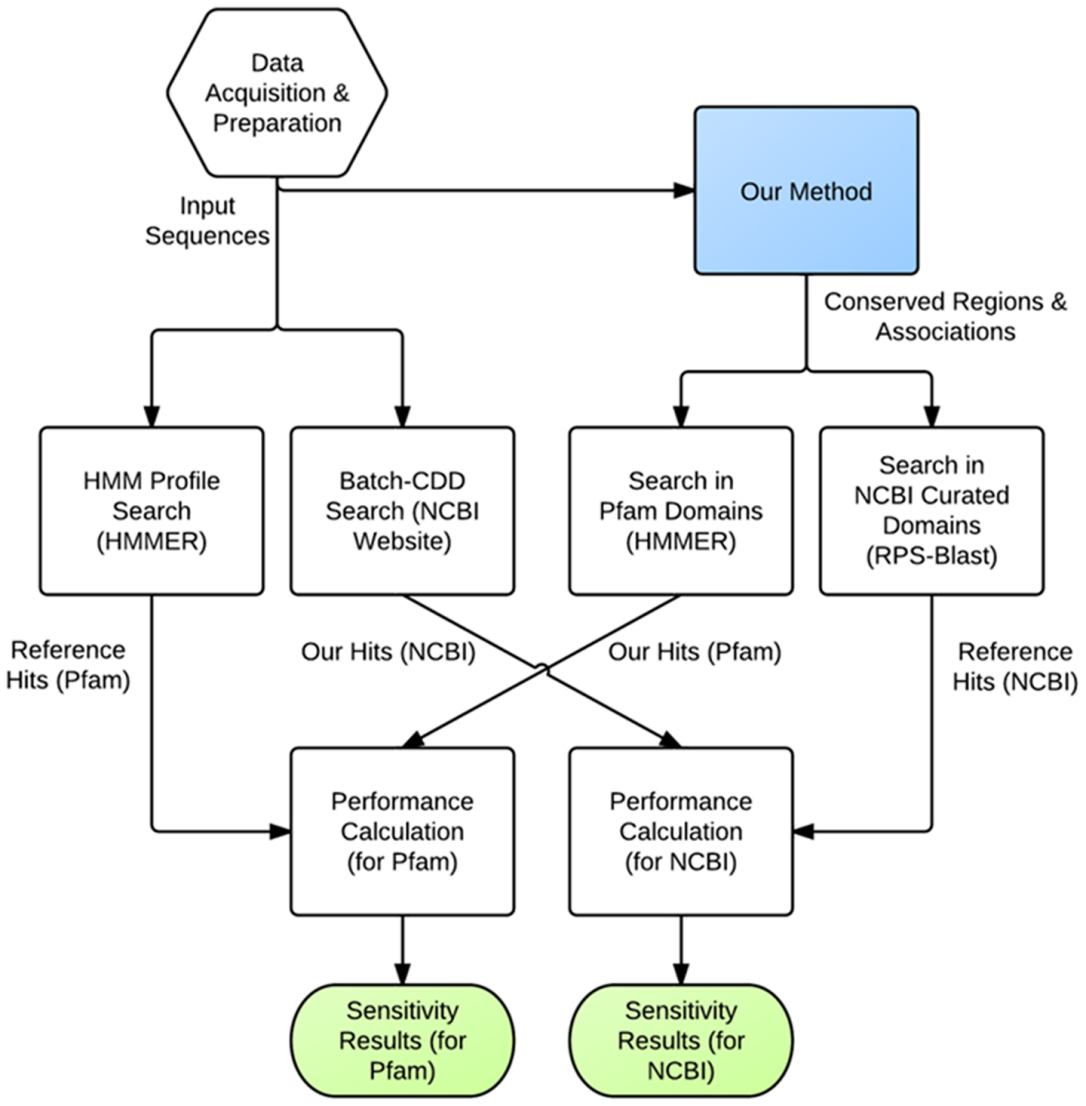
Flow diagram of the performance test. Flow diagram of the performance test for the proposed method in the identification of reference domains in human proteins.

The performance was assessed in terms of the number of accurately detected domains/family regions using the results on the human protein dataset. The test steps are shown in Figure 9 as a flow diagram. In order to generate the reference domain association set, first, the human protein dataset was queried in batch-CDD search tool in NCBI CDD web site with the default parameters using NCBI curated domain profiles as the database and an e-value cut off of 0.05. Next, a standalone version of HMMER v3.0 algorithm [26] was used for querying the dataset through Pfam-A (manually curated) profile Hidden Markov Model database with the default options. This way, we have discovered the confirmed domains on the test sequences separately for Pfam-A and NCBI curated domain databases.

In the following step, the consensus sequences of the identified conserved region profiles were generated in order to search against the pre-formatted domain database of NCBI CDD and profile HMM’s of Pfam. Each conserved region profile consensus sequence and domain profile were aligned to each other and significant matches were identified using the local Reverse Position Specific Blast (Rps-blast) algorithm [27] for NCBI curated domains and HMMER v3.0 for Pfam-A entries with default parameters in both cases. Rps-blast is a blast type algorithm used to search a query sequence against a database of profiles in order to discover significant matches [27]. A significant alignment between a conserved region profile and a reference domain indicated a high likelihood that they represent the same domain. In cases where there were more than one significant hit, the most significant hit -with the lowest E-value- was accepted to represent the corresponding conserved region. In cases with no significant hits to the query profile, those regions were not paired with any reference domains.

The identification performance was measured using sensitivity and precision scores. To this end, the domain hits to the input proteins found by the proposed method were compared to the reference domain hits and the true positive (TP), false negative (FN) and false positive (FP) rates were calculated. A true positive hit was obtained when the same domain was found both by the proposed method and the reference domain search on a protein. When the proposed method failed to find a domain present in reference search, this was counted as a false negative. A false positive was obtained if a hit by the proposed method did not present in the reference pool. The rates represented the average hits for all proteins in the dataset to display a global performance of the proposed method in the identification of domains in human proteins.

**Figure 10:**
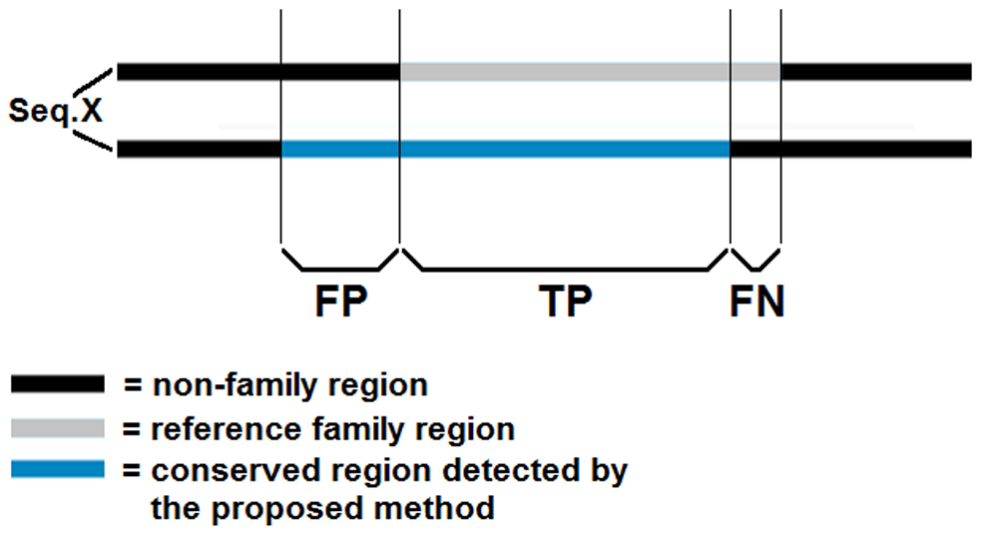
Residue count performance test. Representation of the residue count performance evaluation.

Apart from the count of the recovered documented domains, we have also determined the residue count measure that calculates the overlap of the positions containing domains/family regions between the reference assignments and the positions of the conserved regions identified by the proposed method. For each sequence in the dataset, the positions marked as part of a domain/family region by both the proposed method and the reference Pfam associations were counted as true positives (TP), while those marked by the reference hits but missed by the proposed method were counted as false negatives (FN), and those marked by the proposed method but not in the reference hits as false positives (FP). Figure 10 shows a representative case for the residue count performance evaluation. The rows represent the same sequence (namely, the sequence X). On the top row, the grey region represents a reference family region documented in the Pfam database. The bottom row shows the same sequence with the conserved region detected by the proposed method in blue along with the residues contributing to the TP, FP and FN rates.

## Results

First, reference datasets from SCOP Domain Database [6] were clustered and the performance of the proposed method’s success in separating the protein sequences into families was measured and compared with the conventional methods. Second, the method was applied on a genome wide dataset of human protein sequences to obtain a global familial relation map. The accuracy of the identified relations was evaluated with respect to the current domain assignments in Pfam [3] and NCBI CDD [16] databases. All computations were done using a single core of a system with two quad-core processor operating at 2.3 GHz and 50 GB of RAM.

### Clustering with Reference Datasets

We tested the performance of the proposed method in clustering amino acid sequences using gold standard reference datasets used in previous studies in the literature. Five different datasets from SCOP 1.75 Database [6] previously analyzed by Paccanaro *et al.* and Nepusz *et al.* to test their widely used method Spectral Clustering were taken exactly as they appeared in the referenced studies. The clustering performance was evaluated at the level of superfamilies. Four of these datasets were generated by manually curating domain sequences from different superfamilies in the SCOP 1.75 Database and composed of 550 to 670 sequences each, located in 5 to 6 superfamilies. The fourth dataset was composed of the members from 8 superfamilies and was regarded as a more difficult case for clustering algorithms [13]. The fifth dataset was composed of all domain sequences in SCOP 1.75 Database refined further by removing the sequences with pairwise identity values greater than 95% (ASTRAL-95) via ASTRAL Database [28], and by removing the members of the superfamilies with less than five domains [13]. This final dataset called SCOP_≥5_ contained 14309 sequences from 632 superfamilies and represented one of the most difficult cases known for sequence clustering methods [13]. We have applied the proposed method to these datasets with the default parameters without any specific parameter tuning. The proposed method’s optional final clustering was obtained by incorporating a fast clustering process at the end by a Connected Component Analysis using the correspondence between the conserved regions and the sequences as the input similarity matrix. The total CPU time was around 15 minutes each for the analyses of SCOP datasets 1 through 4 and nearly 12 hours for SCOP dataset 5.

The comparisons of the results with the gold standard were carried out as described in [13] to ensure a fair assessment of the methods. The clustering performance was calculated via the combined F-scores, defined as the combination of precision and recall with equal contributions [12]. The combined F-score was calculated as in [13].

The F-score measures the recognition performance by incorporating both precision and recall scores into a single number [12], [13]. Since the cluster corresponding to a gold standard superfamily was not known in advance, precision and recall scores were calculated for all cluster and superfamily combinations. The combinations that maximized the combined F-score were then selected as matching sequence groups. The calculations of the precision and recall scores as well as the combined F-score are shown in Equations 2, 3 and 4 respectively.
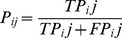
(2)

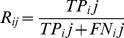
(3)


In the expressions above, *P_ij_* and *R_ij_* are the precision and recall scores respectively for the superfamily *i* and the cluster *j*. *TP_ij_* is the number of proteins both present in the superfamily *i* and the cluster *j*. *FP_ij_* is the number of proteins present in the cluster *j* but not in the superfamily *i*. *FN_ij_* is the number of proteins present in the superfamily *i* but not in the cluster *j*. As for the combined F-score,
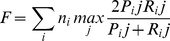
(4)


where *i* indexes the superfamilies and *j* indexes the clusters, *n_i_* is the number of proteins in superfamily *i*, *P_ij_* and *R_ij_* are the precision and recall values respectively.

The clustering performances of previous methods given in [13] are shown in Table 1 with the addition of the proposed method (CRIS) in the last column. The method listed as CCA represents the Connected Component Analysis that is also used as an intermediate step in the proposed method. The others, TribeMCL and Spectral Clustering were described in the Introduction Section.

**Table 1:** Clustering performance results on gold standard datasets from SCOP Database.

	Number of	F-scores			
	sequences	CCA	TribeMCL	SCPS	CRIS
**Dataset 1**	669	0.530	0.630	0.844	**0.866**
**Dataset 2**	587	0.681	0.772	**0.905**	0.884
**Dataset 3**	567	0.588	0.625	0.893	**0.906**
**Dataset 4**	654	0.497	0.573	0.685	**0.740**
**Dataset 5**	14309	0.530	0.576	0.607	**0.641**

CCA: Connected Component Analysis, SCPS: Spectral Clustering.

On the first 3 datasets representing relatively easy clustering instances, the proposed method’s performance was comparable to Spectral Clustering, the top performing algorithm from the literature. On the fourth and fifth datasets, the proposed method outperformed all the alternatives, albeit slightly. This demonstrates the effectiveness of the proposed approach based on statistical grouping over detected conserved regions.

To supplement these results, we have carried out an additional test to verify that the increased performance was not due to the use of Smith-Waterman pairwise alignment in the first step instead of the faster but less accurate Blast algorithm used in Spectral Clustering. To this end, the Blast pairwise alignment results for datasets 1, 2, 3 and 4 were directly taken from [13], and the corresponding e-values were used as input to the proposed method. The results were similar to those obtained before: F-scores of 0.894, 0.864, 0.904 and 0.724 were achieved for datasets 1, 2, 3 and 4 respectively, indicating that the proposed method’s better performance was not due to the use of an optimal pairwise alignment algorithm. In addition, even though these datasets only contained domain sequences from SCOP database, the proposed method extracted the most conserved core regions. As a result, remote sequences were clustered more accurately, owing to the correspondence between the conserved regions and the input samples.

In order to assess the contribution of conserved region merge and refinement step in the clustering performance of the proposed method, the performance in the clustering of SCOP datasets was measured before and after the conserved region merge and refinement step. The results indicated that this step provided an average increase of 28.5% in the clustering performance of the method as measured by the F-measure. Also the difference between the average clustering performance (F-measure) of the initial clustering (using the e-values of all-against-all pairwise alignment, threshold with the selected value: 0.01) and the final result was nearly 18% in favor of the finalized procedure. The reduced performance in the initial clustering was mainly due to the grouping of diverse sequences together at this step.

At this point, it should be noted that the datasets (from SCOP Database) are composed of single domain sequences and not full proteins, and thus, they are depleted of non-domain segments such as low complexity regions. As a result, the measured performance of the method on this dataset may not represent the performance on the datasets composed of full protein sequences. However, the performance of the method in the identification of the documented domains and family regions on a genome wide dataset which will be discussed below represents the potential of the method on full and multi-domain proteins.

### Relational Mapping of Human Proteins and Automatic Domain/Family Region Prediction

Next, we have applied the proposed method to a genome wide collection of human protein sequence data. The reason for choosing a wide range of human proteins was to obtain a global familial relation map of human proteins as well as to determine the performance of the proposed method in predicting domains in a genome-wide collection of protein sequences.

To this end, we have downloaded the accession numbers of human proteins with GO associations from the Gene Ontology Project web site [29]. Gene Ontology is a project aiming to standardize the gene and gene product attributes by assigning controlled vocabulary terms to each entry under three main headings: molecular function, biological process and cellular component [29]. The sequences filtered through the careful inspection of GO ensures a certain degree of reliability in the functional annotations. All of the sequences in the human protein dataset used in this study contained at least one GO association. This way, the newly discovered relations between the sequences can also be tested further regarding their GO associations. Furthermore, sequences with no annotations were not expected to provide useful information to assess the performance of the proposed method to identify documented annotations, and they were subsequently discarded from the analysis.

Protein sequences were downloaded from the UniProt Database [30]. Human protein sequences shorter than 100 amino acids or longer than 10000 amino acids were assumed to be outliers and were also removed from the dataset. Filtering the sequence set based on length was an attempt to improve the statistical analysis. Protein sequence databases contain redundant entries such as the sequence fragments sent by different external sources. The pool of sequences that are shorter than 100 amino acids contain a large amount of these redundant sequence fragments. In a multiple alignment instance, these redundant sequences tend to align with each other and subsequently risk creating false conservations. Furthermore, the probability of non-specific alignment increases with shorter sequences that may also lead to falsely discovered conservations. The final dataset consisted of 17793 human protein sequences. The total CPU time for the analysis of the human protein dataset was approximately 18 hours.

**Figure 11:**
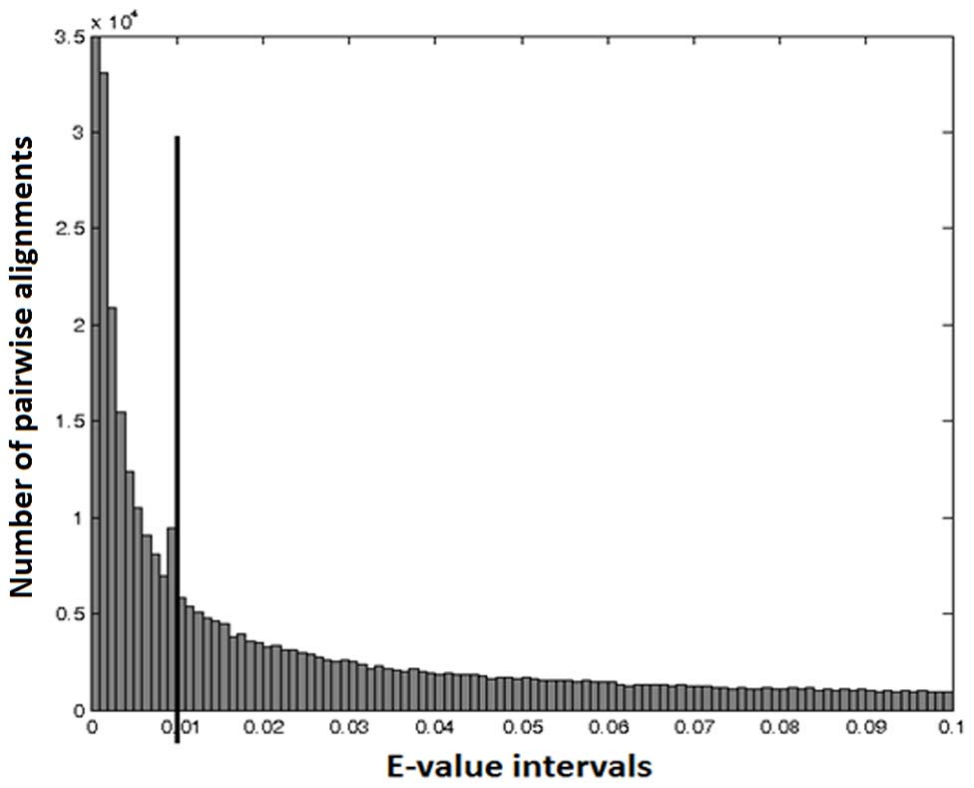
E-value histogram of all-against-all pairwise alignment. E-value histogram of all-against-all pairwise alignment of the human protein dataset.

Following the initial pairwise local alignment and connectivity map thresholding, 3592 connected components were formed of varying sizes along with 2440 singleton components. Figure 11 shows the histogram of the all-against-all pairwise alignment e-values, with the threshold shown by the vertical line. Only the e-value counts between 0 and 0.1 are shown on the figure for readability. Within the non-singleton components, 6537 maximal cliques were located following the elimination of the redundant cliques. After the remaining intermediate steps, a total of 4753 distinct conserved regions were identified and presented in a table showing the association of each conserved region with the input human protein sequences. Conserved regions, in this context, refer to the sequence segments with potential functional and/or evolutionary attributes that correspond strongly to domains and other family regions. The proposed method identified a total of 108588 hits on the human protein dataset, as the total number of associations between the input sequences of 17793 human proteins and the discovered 4753 conserved regions.

Following the application of the method to the human protein dataset, the resulting conserved region profiles were tested for typical sources of non-homologous alignments such as low complexity regions, signal-peptides, coiled-coils and trans-membrane motifs. To this end, NCBI Blast segmasker script [31] was used with the default options to scan for the low complexity regions. Stand-alone SSEARCH algorithm from FASTA v36.3.5 software package [17] was used to align the consensus sequences of the conserved region profiles against signal peptide (http://www.signalpeptide.de/) and trans-membrane motif (PDBTM) [32] databases. A relatively loose e-value cut off of 0.01 was selected to derive all possible alignments. In addition, CCHMM web-server [33] was employed to test the conserved regions against coiled coils. The results are shown in Table 2. The first 4 rows represent the number of conserved regions containing the aforementioned segments; the fifth row shows the average ratio of the corresponding positions to the total number of positions in all conserved regions. Finally, the columns represent different types of the sources of non-homologous alignments. These results indicate that the overlap between the sequence strings causing non-homologous alignments and the output conserved regions of the human protein dataset is considerably low.

**Table 2:** The results of the test for the typical sources of non-homologous alignment of the conserved regions obtained after the human protein dataset analysis.

Ratio of the compositionally biased region:	Type of the region			
	Low complexity	Signal peptide	Trans membrane	Coiled coil
**Number of CR with > 10%**	1225	127	640	24
**Number of CR with > 25%**	434	40	586	17
**Number of CR with > 50%**	81	6	439	10
**Number of CR with > 90%**	8	0	127	2
**Total residue count (%)**	0.072	0.007	0.090	0.067

CR: Conserved regions.

In order to validate the results on human proteins further, we have evaluated the correspondence between the conserved regions identified above and the Pfam-A domains in Protein Families Database 26.0 [3] as well as the NCBI curated domains in Conserved Domain Database v3.07 [16]. A total of 674 human protein sequences in Pfam-A and 171 sequences in NCBI CDD were found to contain more than 6 significant domain hits. Due to the high number of hits these sequences were removed from the performance tests as outliers. The analyses were thus carried out on the proteins with 6 domain hits or less on different regions of the sequences -not counting multiple hits on a particular region-.

HMM profile search identified a total of 24197 reference Pfam-A domain type hits on our dataset, while Batch-CDD search in NCBI CDD web site identified 16526 reference domain hits. The total number of reference Pfam hits (considering domain, family, repeat and motif type hits) on human proteins were 41551. 45% of the 17793 human proteins lacked domain type assignments in Pfam database, while this number was at 47% for the NCBI CDD. Considering all four types of entries in Pfam database, only 9% of the human protein sequences were found to lack any assignments. Domain type entries in Pfam database are usually more reliable than the other types since they are verified by additional sources such as structural data where available [34]. As a result, we calculated the performance for the domain type hits separately from the rest.

Table 3 shows the sensitivity scores observed in the identification of the domain type hits in Pfam in human protein sequences. The scores were 0.744 for Pfam and 0.776 for NCBI CDD. Nearly 76% of the domain hits on the reference databases were accurately recovered by the proposed method, with more than 77% and 61% of the reference domains on Pfam and NCBI CDD respectively being from multi-domain proteins. Table 4 shows the sensitivity and precision scores for the mixed type hits (domain, family, repeat and motif) in the Pfam database. The precision scores here can be deceptive since it incorporates the false positives count as well. In our case, these hits were ambiguous as they could be true false positives or new family regions undiscovered so far. Nevertheless, precision was calculated to obtain a rough indication about how often the proposed method returns an incorrect assignment. Note that the precision score was calculated only for protein sequences that had at least one reference Pfam hit.

**Table 3:** Performance of the proposed method in identifying reference domains in the human protein dataset.

Number of domains in	Sensitivity values	
reference database:	Pfam ref.	NCBI CDD ref.
**Single domain:**	0.809	0.919
**Up to 2 domains:**	0.779	0.883
**Up to 3 domains:**	0.765	0.862
**Total:**	0.744	0.776

(Sensitivity: TP/(TP + FN), TP: true positives, FN: false negatives).

**Table 4:** Performance of the method (hit counts) in the identification of the reference (all types) Pfam hits on the human proteins.

Number of hits in Pfam database:	Performance results	
	Sensitivity	Precision
**Single domain:**	0.754	0.522
**Up to 2 domains:**	0.684	0.522
**Up to 3 domains:**	0.663	0.504
**Total:**	0.631	0.505

Following the domain/family region counts, the residue counts were also considered as another performance metric as it can provide additional insight regarding the performance of the method in identifying domains/family regions. The sensitivity and precision scores for the residue counts with respect to the reference mixed type Pfam hits were calculated, though it should be noted that precision here again can be deceptive (Table 5).

**Table 5:** Performance of the method (residue counts) in the identification of the reference (all types) Pfam hits on the human proteins.

Number of hits in Pfam database:	Performance results		
	Sensitivity	Precision	
**Single domain:**	0.739	0.654	
**Up to 2 domains:**	0.731	0.644	
**Up to 3 domains:**	0.733	0.636	
**Total:**	0.739	0.628	

(Sensitivity: TP/(TP + FN), TP: true positives, FN: false negatives).

(Precision : TP/(TP + FP), TP: true positives, FP: false positives).

During the performance evaluation of the proposed method, the recovered conserved regions agreed to a significant degree with the reference curated domains/family regions. However, a significant portion of the conserved regions could not be paired with any of the documented domains and were therefore proposed as novel conserved regions. Table 6 shows the statistics on the known and the novel conserved regions against the reference domains in Pfam-A and NCBI CDD databases. The first column shows the number of the known conserved regions, while the second column shows the novel ones, out of the 4753 recovered regions. Approximately 51% and 41% of the conserved regions did not have a correspondence on the Pfam-A and NCBI CDD databases respectively.

**Table 6:** The statistics on the conserved region pairings with the reference hits.

	Number of conserved regions:	
	match	no-match
**Pfam-A**	2324	2429
**NCBI CDD**	2795	1958

Out of 4753 conserved regions.

In order to determine if these novel conserved regions corresponded to the automatically generated low significance domain entries in the Pfam database, we queried the conserved region profiles against a database containing both Pfam-A and Pfam-B [3] entries. Pfam-B entries were generated to supplement the Pfam database for the sequences where there are no Pfam-A associations (Finn et al., 2010). Pfam-B was generated automatically using the ADDA algorithm (Heger and Holm, 2003) that finds appropriate sites for domain boundaries and cuts the sequences from these positions to call one side of the boundary as domain 1 and the other as domain 2. As a result, all of the residues in a protein sequence reside in a domain. Note that the logic behind this method is different than the proposed method that only marks the conserved regions as potential domains or family regions. The results showed that only 27% of the novel conserved regions corresponded to Pfam-B domains, meaning that nearly 73% of them were indeed novel domain or family region candidates.

Finally, we have carried out a manual analysis on the conserved regions identified on the human protein dataset for possible new functional associations. Firstly, we noted that the 1009^th^ conserved region largely overlapped with the proteins annotated with the term: GO:0008270 – zinc ion binding, with 508 out of 513 proteins containing this conserved region being associated with this GO category. In other words, more than 99% of the proteins in the corresponding cluster had zinc ion binding associations. One of the five proteins that lacked this particular GO association, *Kelch-like ECH-associated protein 1* (KEAP1) (UniProt identifier: ‘Q14145’) takes part in the suppression of the transcriptional activity of NFE2L2/NRF2 protein by targeting it for ubiquitination and degradation by the proteasome [35]. This protein has only one GO molecular function association (GO:0005515 – protein binding) and it has no direct ancestor-child relation to GO:0008270. They are joined at a high level on the hierarchical GO tree at the category GO:0005488 - binding. As a result, there appears to be no indication that this protein has a documented zinc ion binding function association in GO. Due to the high correlation between the GO:0008270 term and the conserved region 1009, our results predict that this protein have a zinc ion binding function. To test this prediction, the amino acid sequence of this protein was searched in the Pfam database. One of the three types of structural domains was “BTB/POZ domain” (PF00651), found frequently in zinc finger proteins [36]. This finding supports our prediction since zinc ion binding function is naturally associated with zinc finger proteins. In order to take another look at the case, we have analyzed the other conserved regions residing on this protein and found that the domain *Zinc finger, C2H2 type* (PF00096) in Pfam was associated with one of these conserved regions with high significance. This domain, in turn, is also associated to GO:0008270 though not to GO:0005515, providing extra support in favor of this prediction.

Furthermore, the location of the 1009^th^ conserved region along the amino acid sequence of this protein was between the positions 310 to 342. In Pfam database this region overlapped with a Kelsh motif (PF01344) between the positions 317 to 359. The alignment of the Kelsh motif with this region gave a bit score of 21.9. The 1009^th^ conserved region was aligned to this region with a bit score of 45.6 and a similarity of 80% even though the size of the conserved region was significantly shorter than the Kelsh motif: The 1009^th^ conserved region was 38 residues long, while the Kelsh motif contained 47 residues. Finally, a search on the associations between the Pfam hits to KEAP1 and GO terms via pfam2go at InterPro database [4] determined that these Pfam hits were associated only with the category GO:0005515 – protein binding. As a result, the zinc ion binding (GO:0008270) association is indeed a novel finding.

Note that in this analysis, we started from GO associations and predicted a new functional assignment to a protein using the results obtained with the proposed method. Similar analyses can be made on other proteins in the dataset to predict additional novel functional assignments.

## Discussion

In this study, we proposed CRIS: a computational method to identify family relations between protein sequences in diverse datasets over evolutionary conserved regions. In the experiment results, these conserved regions corresponded to the documented structural domains. The identification of these regions was achieved in a completely unsupervised manner using only sequence data that was subjected to pairwise sequence alignment, residue conservation scoring and graph theoretical analyses. In the validation experiments, the method was first applied on gold standard datasets and the functional clustering performance was measured and compared with the conventional methods. The results indicated highly accurate clustering. Second, we used the proposed method to process a genome-wide dataset composed of 17793 human protein sequences to obtain a global familial relation map. As a result, we obtained a table representing the correspondence between the proteins and the recovered conserved regions. Familial relations of the proteins were clearly observed through the connections over these regions. We also measured the correspondence of these conserved regions to the manually curated domain assignments on these proteins both in Pfam and NCBI CDD databases. The results showed that most of the known structural domains were correctly identified even on multi-domain human proteins.

As is well known, grouping amino acid sequences using a linkage method such as a Connected Component Analysis imposes a domain chaining problem: A given sequence pair within a component may not necessarily share a significant sequential similarity, but appear in the same component due to the chain effect where they may both possess similarity to a third sequence over different regions [15], [37]. As a result, being in the same component does not stipulate a shared feature between all sequences in the component, though appearing in different components guarantees the absence of any significant shared features. All shared features, however, are to be discovered within each component. In this work, the detection of the maximal cliques within each connected component was used to discover this mutuality. All sequences residing in the same maximal clique were thus guaranteed to share at least one significant sequential regional similarity, on top of any additional features shared between a smaller number of sequences in the clique.

At this point, component formation procedure may appear to be dispensable since maximal clique search discovers the shared sequence features, but it is important to note that maximal clique finding is an NP-hard problem and it may require a substantial processing even for a relatively small dataset of 500 sequences with an average computational power. Pre-processing with connected component identification ensures the separation of sequence clusters with no inter cluster relationships. In the proposed method, to further reduce the computational load associated with clique identification in large components, groups of 100 random sequences were subjected to maximal clique finding separately. The additional amount of redundancy in the identified maximal cliques was resolved later at the conserved region merge and modification step.

Generally speaking, highly conserved regions along amino acid sequences often correspond to zones with evolutionary and/or functional signatures. Thus, the conserved regions found by the proposed method were expected to capture the known domains in the input sequences. In the results on human proteins, conserved regions did indeed contain domains, with slight variations in detection performance between Pfam and NCBI CDD domains. It is, however, likely that this performance gap was due to the differences in the domain assignments in these databases. It is shown on Table 4 and Table 5 that, as the number of domains in sequences increase, the sensitivity of our method in terms of hit counts decreases whereas the sensitivity in terms of residue coverage remains almost the same. This can be attributed to the ability of the proposed method to capture these regions with a relatively stable performance though it becomes harder to separate them into individual domains/family regions as the number of these domains increase on the sequences. The precision score was also stable and did not change significantly with varying number of reference hits.

As shown in Table 6, nearly half of the recovered conserved regions identified on the human protein dataset did not correspond to the documented domains on the online databases, with only 27% corresponding to the domain entries in the low significance Pfam-B database. The remaining conserved regions can thus be predicted to depict new families that have not been discovered and/or documented so far. The only way to verify these predictions may be detailed studies directed to each sequence individually including experimental work.

Comparative performance evaluation results showed that the proposed method performed better with single domain proteins, and the performance decreased as the proteins with higher number of domains were included. Further inspection of the results revealed that most of the false negative hits belonged to the consecutively located domains on multi-domain proteins. However, when the variety of domain distribution on sequences was large, these domains were identified accurately. This is especially important to be able to identify the domains in multi-domain proteins. During the statistical grouping step, the sequence clusters, each sharing a unique conserved feature were generated. Note that when the variety of domain distribution is sufficiently large, it is possible to construct a new cluster for each unique feature. For very small datasets, however, it becomes considerably harder to distinguish the shared features from a statistical point of view. This makes the proposed method a suitable candidate to analyze shared features on whole proteomes.

On another note, the input sequences that do not align with any other sequences in similarity based sequence analysis methods are generally left out of the results. In our method, we have incorporated the singleton sequences into the analysis by searching for the conserved region profiles once more through all sequences. Owing to the remote homology recognition ability of profile alignments, features hidden inside these sequences have been discovered more clearly. This way, the reference domains were indeed identified on some of the singleton human protein sequences.

At this point, it should be noted that it is not possible to be absolutely certain about the performance of the method in the detection of families and domains on protein sequences without testing on gold standard datasets incorporating established true negative families. One option would be using simulated datasets where the families, domains, evolutionary relations and so forth are known beforehand. However, because of the complexity of the biological sequences and their evolutionary relations, and due to the high number of parameters to be controlled, it is quite difficult to generate realistic simulations. Furthermore, the results obtained on such synthetic datasets would be highly dependent to the properties of the underlying simulation procedures, and defeat the purpose of carrying out performance evaluations to predict future performance on real sequence datasets. Under these circumstances, performance evaluation analyses incorporating simulated sequence datasets were left out of the current study.

It is also important to note that the proposed method is not optimized in any way to outperform its predecessors in computational performance using the specifics of the reference datasets. The principal contribution of this method comes from the improved prediction performances without any guided parameter tuning. As shown in both the clustering analyses and the identification of the regions containing family signatures, the method outperformed its predecessors wherever a comparison was possible.

On a final note, the correspondence table generated by the proposed method provides the associations between the proteins and the conserved regions. As such, it allows inferring protein families as those sharing the same set of conserved regions. However, it can also be viewed to document the relationship between the conserved regions over the proteins that possess them simultaneously. This suggests a duality in the analysis of protein sequences: Just as families of proteins associated with similar functional or structural attributes, one can also consider families of conserved regions that followed through the process of molecular evolution together. As a future work, this duality can be explored and exploited to aid the construction of a parallel analysis of the evolution of whole proteomes. Another potential future study would be the inspection of the correlation between the protein-protein interactions and the associations of the interacting proteins over the conserved regions. Since these regions correspond to highly conserved sequence segments with possible functional signatures, there is a high likelihood that the interactions between the proteins are occurring over these regions.

The proposed method CRIS (Conserved Region Identification and Search) is freely available for academic use as a MATLAB implementation together with the datasets and the results figuring in this article (including the global familial relation map of human proteins) at http://biplab.eee.iyte.edu.tr/en/projects/conregidase/.
